# PD-1-siRNA delivered by attenuated *Salmonella* enhances the antimelanoma effect of pimozide

**DOI:** 10.1038/s41419-019-1418-3

**Published:** 2019-02-18

**Authors:** Tiesuo Zhao, Tian Wei, Jing Guo, Yangeng Wang, Xiangyi Shi, Sheng Guo, Xiaolong Jia, Huijie Jia, Zhiwei Feng

**Affiliations:** 1 Department of Immunology, Xinxiang Medical University, Xinxiang, Henan 453000 People’s Republic of China; 2 Institute of Precision Medicine, Xinxiang Medical University, Xinxiang, Henan 453000 People’s Republic of China; 3 Xinxiang Key Laboratory of Tumor Vaccine and Immunotherapy, Xinxiang Medical University, Xinxiang, Henan 453000 People’s Republic of China; 4 Henan Key Laboratory of Immunology and Targeted Therapy, Xinxiang Medical University, Xinxiang, Henan 453000 People’s Republic of China; 5 Department of Pathogen Biology, College of Basic Medical Sciences, China Medical University, Shenyang, 110001 People’s Republic of China; 6 Department of Pathology, Xinxiang Medical University, Xinxiang, Henan 453000 People’s Republic of China

## Abstract

Melanoma is one of the most aggressive skin cancers worldwide. Although there has been much effort toward improving treatment options over the past few years, there remains an urgent need for effective therapy. Immunotherapy combined with chemotherapy has shown great promise in clinical trials. Here, we studied the cooperative effects of the small molecule drug pimozide, which has a therapeutic effect in melanoma, and RNA interference (RNAi) targeting PD-1, an important immune checkpoint molecule involved in tumor immune escape. PD-1 siRNA was delivered by attenuated *Salmonella* to melanoma-bearing mice in combination with pimozide. Our results demonstrated that the combination therapy had the optimal therapeutic effect on melanoma. The mechanisms underlying the efficacy involved the induction of apoptosis and an enhanced immune response. This study suggests that immunotherapy based on PD-1 inhibition combined with anticancer drugs could be a promising clinical strategy for the treatment of melanoma.

## Introduction

Metastatic melanoma is one of the most aggressive skin cancers worldwide, and there is no effective treatment currently^1^. Surgical resection remains the cornerstone of curative treatment at the early stages of the disease but offers only a small chance for curing metastatic melanoma. The addition of radiotherapy and chemotherapy is not effective^2^. As a result, the prognosis of metastatic melanoma is poor, with an average survival time of less than 1 year^3^. Therefore, more effective treatment strategies for melanoma are urgently required. Pimozide, a Food and Drug Administration (FDA)-approved psychiatric drug and effective dopamine antagonist, was first administered to patients with metastatic melanoma as early as 1979^4^. Previous studies by us and other researchers have shown that pimozide has certain therapeutic effects on melanoma^5,6^. Although favorable responses have been documented, the therapeutic effect must be further improved. Recent studies revealed a promising strategy of combining immunotherapy with chemotherapy, which may further improve cancer treatment.

Immunotherapy has been successfully applied to the treatment of several human cancers^7^. The blockade of immune checkpoints, a newly emerging idea in antitumor immunotherapy, has exhibited curative effects and thus has potential as a new way to cure cancer^8,9^. Programmed death 1 (PD-1) is an important immune checkpoint molecule that can enable tumor cells to escape the host immune response through the suppression of effector T-cell function and the induction of T-cell exhaustion^10^. In addition, multiple basic research and clinical studies have demonstrated that PD-1 blockade can markedly inhibit tumor progression and improve the prognosis of patients with a variety of advanced cancers, including melanoma^11–13^, ovarian cancer^14^, gastric cancer^15^, renal cell cancer^16^, and nonsmall cell lung cancer^17^. These studies have highlighted that anti-PD-1 therapy holds great promise for the treatment of human malignancies. Currently, PD-1 monoclonal antibodies are widely used in the treatment of various malignancies; however, they are expensive and cause side effects, such as autoimmune diseases. Therefore, we applied RNA interference (RNAi) to inhibit PD-1 to effectively evoke immune responses.

A major challenge for tumor gene therapy is choosing an efficient gene delivery system that selectively targets tumors. Several bacteria offer promise as gene therapy vectors, and among them, genetically attenuated *Salmonella* has been widely investigated^18^ and used as a vehicle to deliver plasmids carrying small hairpin RNA (shRNA) to various tumors, including cervical cancer^19^, breast cancer^20^, pancreatic cancer^21^, stomach cancer^22^, ovarian cancer^23^, lung cancer^24^ and prostate cancer^25^. As a facultative anaerobe, *Salmonella* was shown to target hypoxic regions in tumors and preferentially accumulated in tumors compared to normal tissues^26^. In addition to tumor targeting, there are many other benefits of using *Salmonella* for cancer gene therapy, such as its ability to act as an immunostimulant and the low cost^27,28^. Our previous study demonstrated that phoP/phoQ-deleted *S. Typhimurium Salmonella* can efficiently deliver stat3-shRNA into tumor tissues and shows therapeutic effects on hepatocellular carcinoma^29^.

Here, we tested the hypothesis that PD-1 knockdown using small interfering RNA (siRNA) gene therapy delivered by attenuated *Salmonella* is a promising strategy for tumor immunotherapy. We further investigated the antitumor effect of the combination treatment of pimozide with PD-1 knockdown by attenuated *Salmonella* in a mouse xenograft model of melanoma. Our results demonstrated that PD-1 knockdown by siRNA delivered by attenuated *Salmonella* is an effective strategy to induce tumor immunity and suppress melanoma growth. In addition, the melanoma treatment efficacy was greatly enhanced by combining PD-1 siRNA with the anticancer drug pimozide compared with either reagent alone. Moreover, the optimal antitumor effect was achieved by the accumulation of attenuated *Salmonella* in tumor tissue, the inhibition of PD-1 expression, the induction of apoptosis, and the enhancement of immune function.

## Results

### PD-1 siRNA constructs specifically reduced PD-1 expression in EL4 cells

**Fig. 1:**
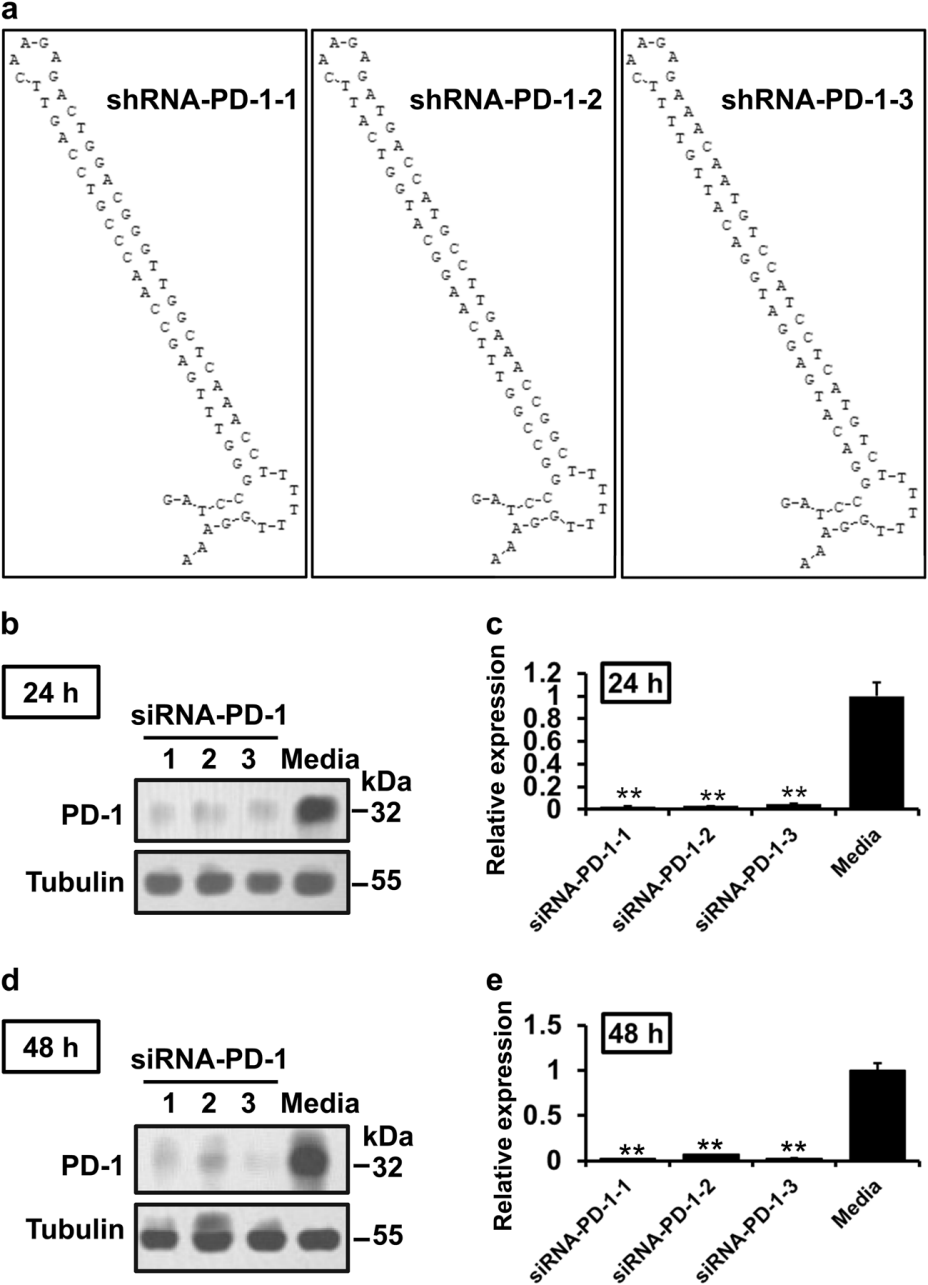
Construction and identification of the recombinant plasmids. **a** Local secondary structure of programmed death 1 (PD-1) mRNA at the regions targeted by the three PD-1 small interfering RNAs (siRNAs) synthesized in the study. **b** Effect of three plasmids containing different sequences of PD-1-specific short hairpin RNA (shRNA) on EL4 cells for 24 h. **c** Quantification of PD-1 protein levels in (**b**) from three independent experiments. **d** Effect of three plasmids containing different sequences of PD-1-specific shRNA on EL4 cells for 48 h. **e** Quantification of PD-1 protein levels in (**d**) from three independent experiments. Media indicate untreated cells. The data are presented as the mean ± standard deviation (SD). **P < 0.01 vs the media group

Based on siRNA design principles, we designed three different PD-1 siRNA sequences and inserted them into the pSilencer plasmid as described previously^29^. The three plasmid vectors expressing PD-1-specific siRNA were named pSi-PD-1-1, pSi-PD-1-2, and pSi-PD-1-3 (Fig. 1a). The construction of these plasmids was successful, as confirmed by enzyme digestion and sequence analysis (data not shown). To determine the effect of the three shRNA expression plasmids, we transfected them into EL4 cells and detected PD-1 expression at 24 and 48 h by western blotting (WB). The results showed that PD-1 expression was significantly decreased in pSi-PD-1-transfected cells after 24 and 48 h compared to control cells (media alone) (Fig. 1b–e, *P* *<* 0.01), while the most significant inhibition of PD-1 expression was detected in the pSi-PD-1-1 group. Figure 1d, e shows a similar pattern to Fig. 1b, c, indicating a statistically significant decrease in expression (*P* < 0.01). Therefore, we used the plasmid pSi-PD-1-1, which harbors shRNA-PD-1-1, for subsequent experiments.

### Attenuated *Salmonella* preferably accumulated in tumors

**Fig. 2:**
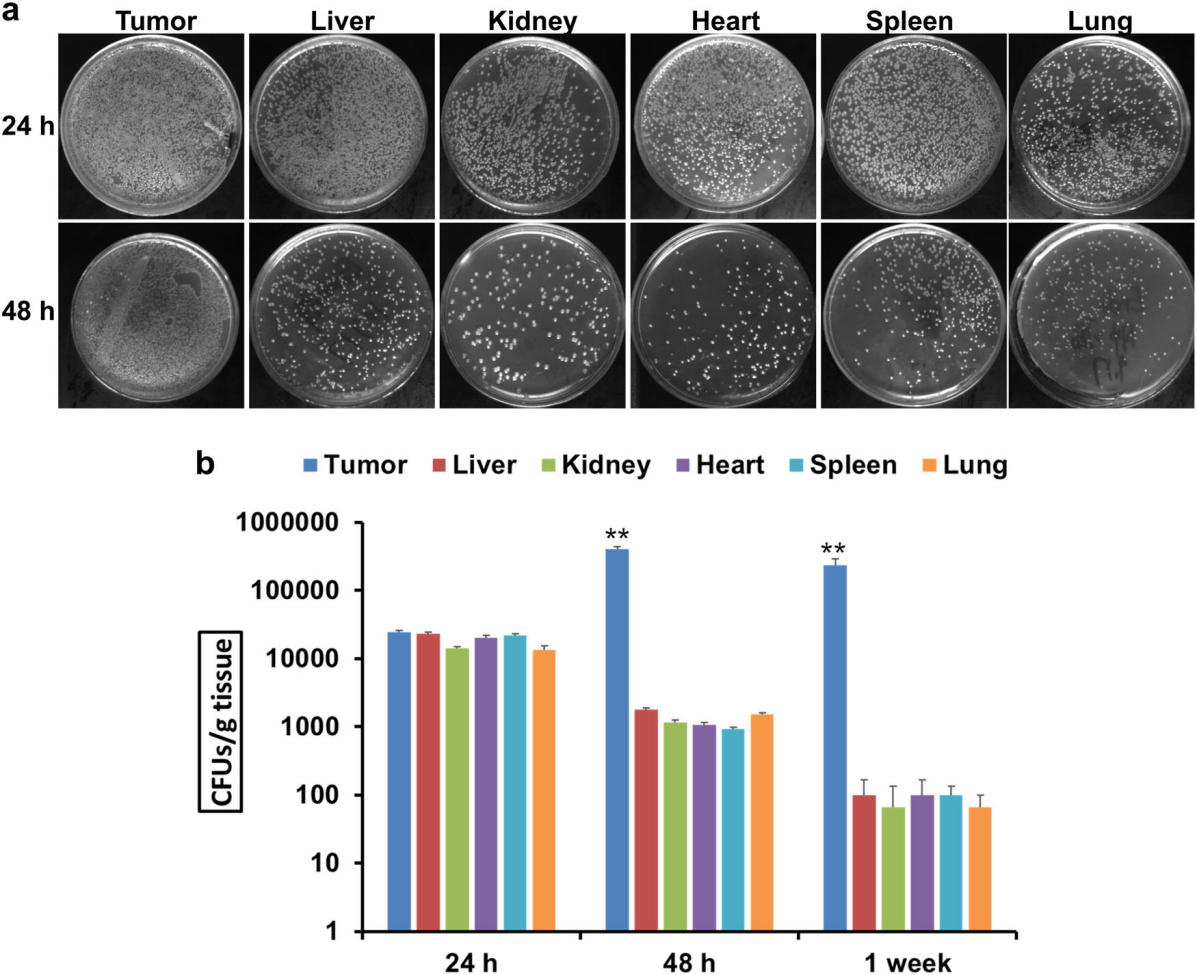
Attenuated *Salmonella* distribution in tumors and other organs. At 24 h, 48 h, and 1 week after intraperitoneal (i.p.) injection of attenuated *Salmonella*, the mice were killed, and the tumors, liver, spleen, lungs, heart, and kidneys were resected, homogenized, diluted, and plated onto LB agar plates. **a** Representative images of LB agar plates. **b** Quantitative analyses of bacterial counts at 24 h, 48 h, and 1 week post injection. The data are presented as the mean ± SD of three separate experiments. **P < 0.01 vs the tumor group

To ensure that the attenuated *S. Typhimurium Salmonella* strain transformed with the siRNA-PD-1 expression plasmid preferentially accumulated in tumor tissue, the distribution of bacteria in B16 xenografts and major organs (liver, spleen, lung, heart and kidney) of mice that received an intraperitoneal (i.p.) injection of attenuated *Salmonella* carrying pSi-PD-1 was monitored. The number of bacteria was almost the same in tumors as in other organs at 24 h post injection but was significantly higher in tumors than in other tissues at 48 h (Fig. 2a, b). Quantitative analyses revealed that after injection, the bacteria gradually accumulated in tumors rather than in other organs at a ratio above 1000:1 (Fig. 2a, b, *P* < 0.01). Only a small amount of *Salmonella* was detected in the heart, liver, lungs, spleen, and kidney 1 week after administration, while significant amounts of *Salmonella* remained in tumor tissues (Fig. 2b, *P* < 0.01). At 3 weeks, almost no *Salmonella* colonies were found on plates from all organs and tumors, indicating that the bacteria had been nearly completely cleared by the body (data not shown).

### Combined treatment with pimozide and pSi-PD-1 showed remarkable antitumor activity in a melanoma xenograft model

**Fig. 3:**
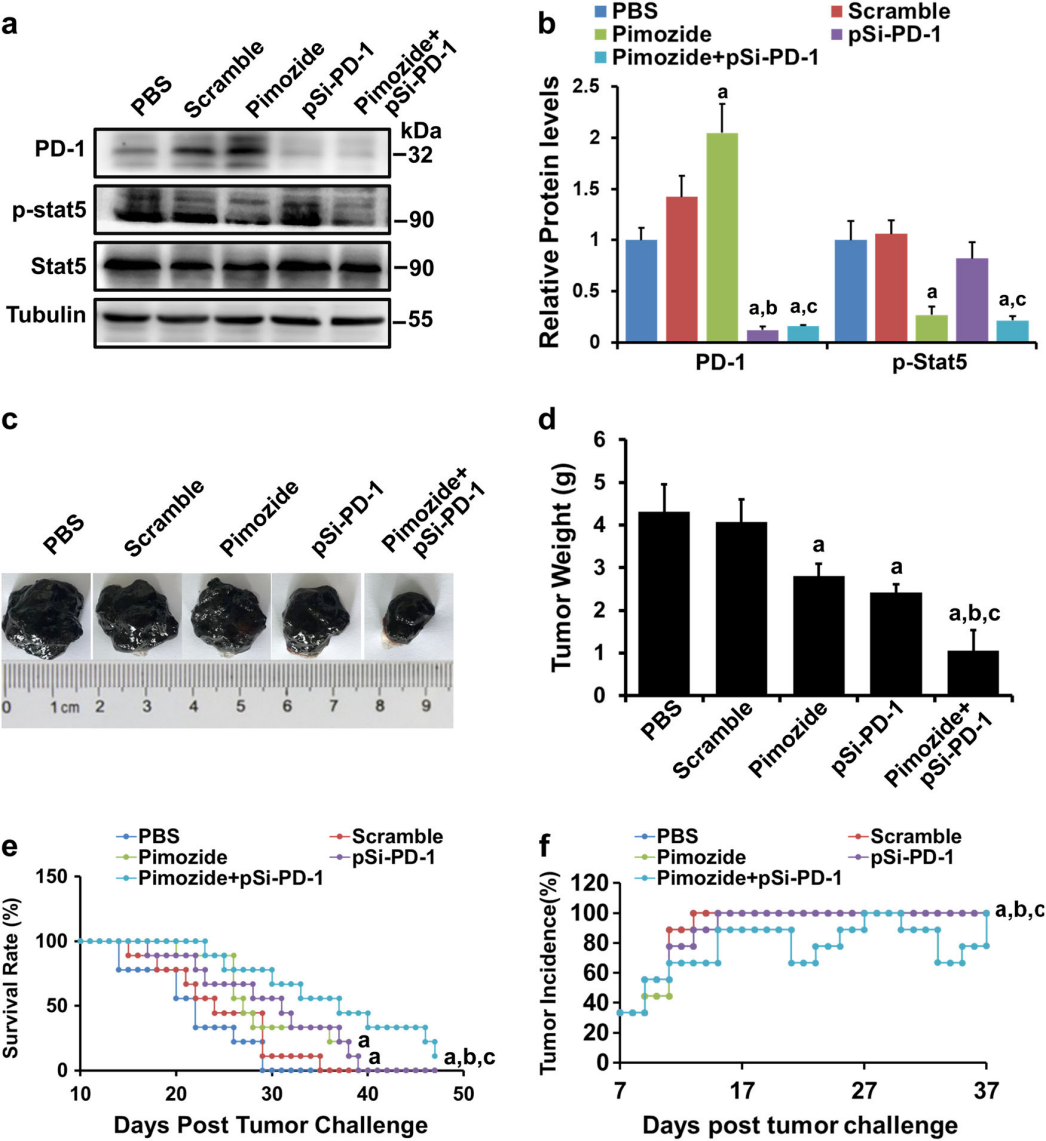
Antitumor effects of various treatments in vivo. At day 14 after tumor implantation, the mice were treated with various treatments. **a** Analyses of programmed death 1 (PD-1), p-Stat5, and Stat5 expression using western blotting (WB). **b** Semiquantitative analyses of relative protein levels. **c** Images of representative tumors from every group. **d** Average tumor weight of each group. **e** Survival curves. **f** Tumor incidence curves for each group. ^a^*P* < 0.05 vs the Mock or pSi-Scramble group. ^b^*P* < 0.05 vs the pimozide group. ^c^*P* < 0.05 vs the pSi-PD-1 group

To explore whether PD-1 inhibition could improve the antitumor effects of pimozide in B16 tumor-bearing mice, these mice were administered pimozide, pSi-PD-1, or the combination of pimozide and pSi-PD-1. As shown in Fig. 3a, b, PD-1 expression was considerably increased in the pimozide treatment group but was decreased in the groups treated with pSi-PD-1 alone or the combination of pimozide and pSi-PD-1. As Stat5 is the target of pimozide, we further detected p-Stat5 and Stat5 levels in tumors from the various groups. The results indicated that p-Stat5 protein levels were lower in the pimozide and combination treatment groups than in the other three groups. In addition, tumor size and weight were markedly reduced in the pimozide, pSi-PD-1, and combination treatment groups compared to the phosphate-buffered saline (PBS) or pSi-Scramble group. However, the smallest tumors were observed in the combination group (Fig. 3c, d). Survival assays showed that compared with PBS or pSi-Scramble, treatment with pSi-PD-1 or pimozide significantly prolonged the survival of B16 tumor-bearing mice (*P* < 0.01, Fig. 3e). Notably, the combination of pSi-PD-1 and pimozide remarkably prolonged the survival rate compared with all other treatments (*P* < 0.01, Fig. 3e). Importantly, the combination treatment group exhibited the lowest tumor incidence among all the groups. Although two mice in the combination treatment group showed complete tumor regression during the treatment period, the tumors returned 2 days later. One of the mice in the combination treatment group was alive at the end of the observation period.

### Cotreatment with pimozide and pSi-PD-1 induced the apoptosis of B16 tumor cells

**Fig. 4:**
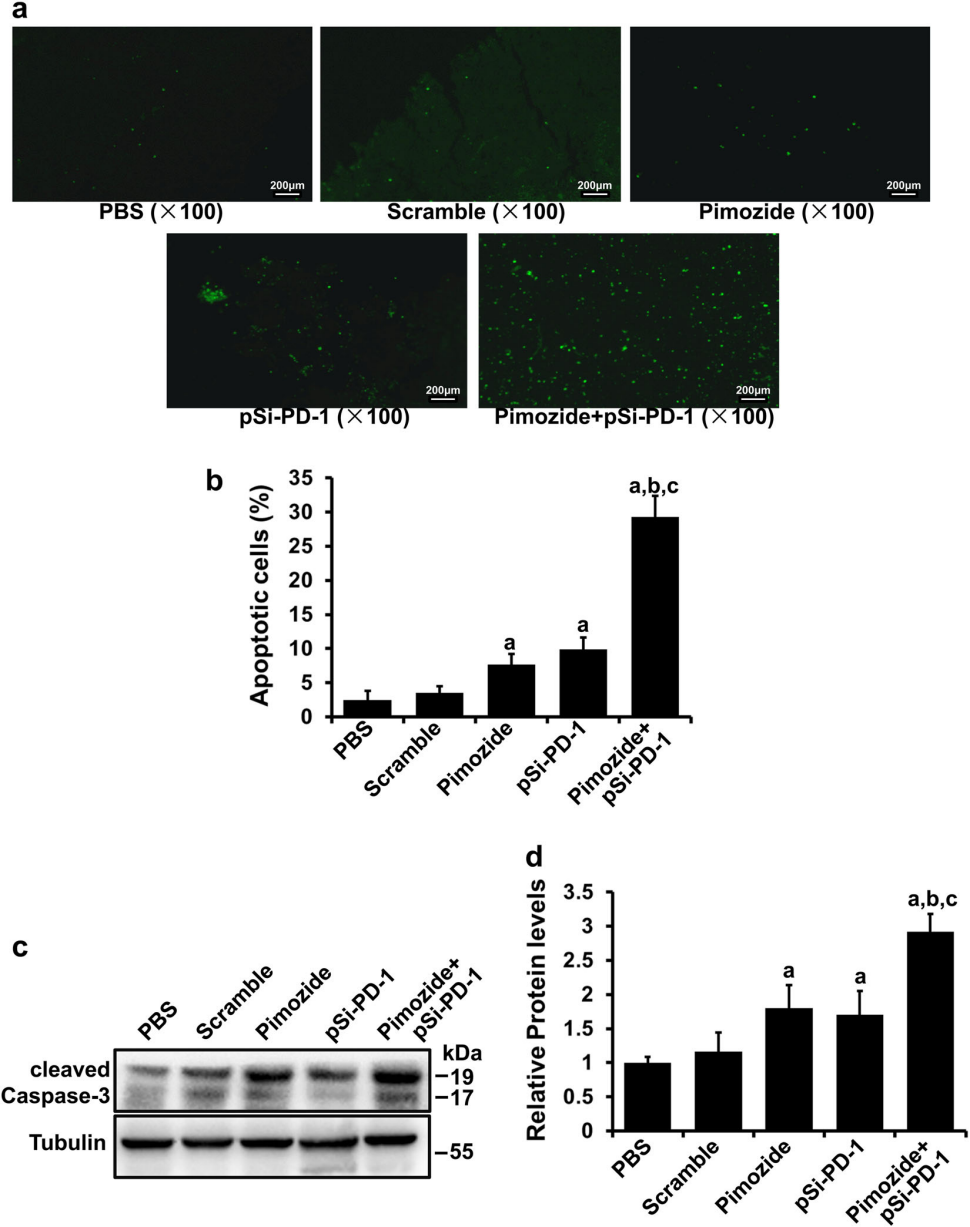
Effect of cotreatment on apoptosis in vivo. **a** Apoptosis of tumor cells in each group detected by TUNEL (terminal deoxynucleotidyl transferase dUTP nick end-labeling) staining. **b** Quantitative analysis of apoptotic cells in TUNEL assays. **c** Cleaved caspase 3 levels were examined by western blotting (WB) in B16 xenografts exposed to various treatments. **d** Semiquantitative analysis of relative protein levels. ^a^*P* < 0.05 vs the Mock or pSi-Scramble group. ^b^*P* *<* 0.05 vs the pimozide group. ^c^*P* < 0.05 vs the pSi-PD-1 group

To characterize the mechanisms underlying the antitumor effects of the combination therapy, we first determined the effect of cotreatment on tumor apoptosis using TUNEL (terminal deoxynucleotidyl transferase dUTP nick end-labeling) assays and WB. The levels of cleaved caspase 3 were analyzed following various treatments. TUNEL staining revealed an increase in the number of tumor cells undergoing apoptosis in the pimozide, pSi-PD-1, and cotreatment groups, with the largest increase in the cotreatment group among all the groups (Fig. 4a, b, *P* < 0.01). Moreover, Fig. 4c, d shows that cleaved caspase 3 levels were greatly increased in the pimozide and combination treatment groups, with a particular increase in the combination treatment group (*P* < 0.01).

### Combined treatment with pimozide and pSi-PD-1 increased the recruitment of T lymphocytes

**Fig. 5:**
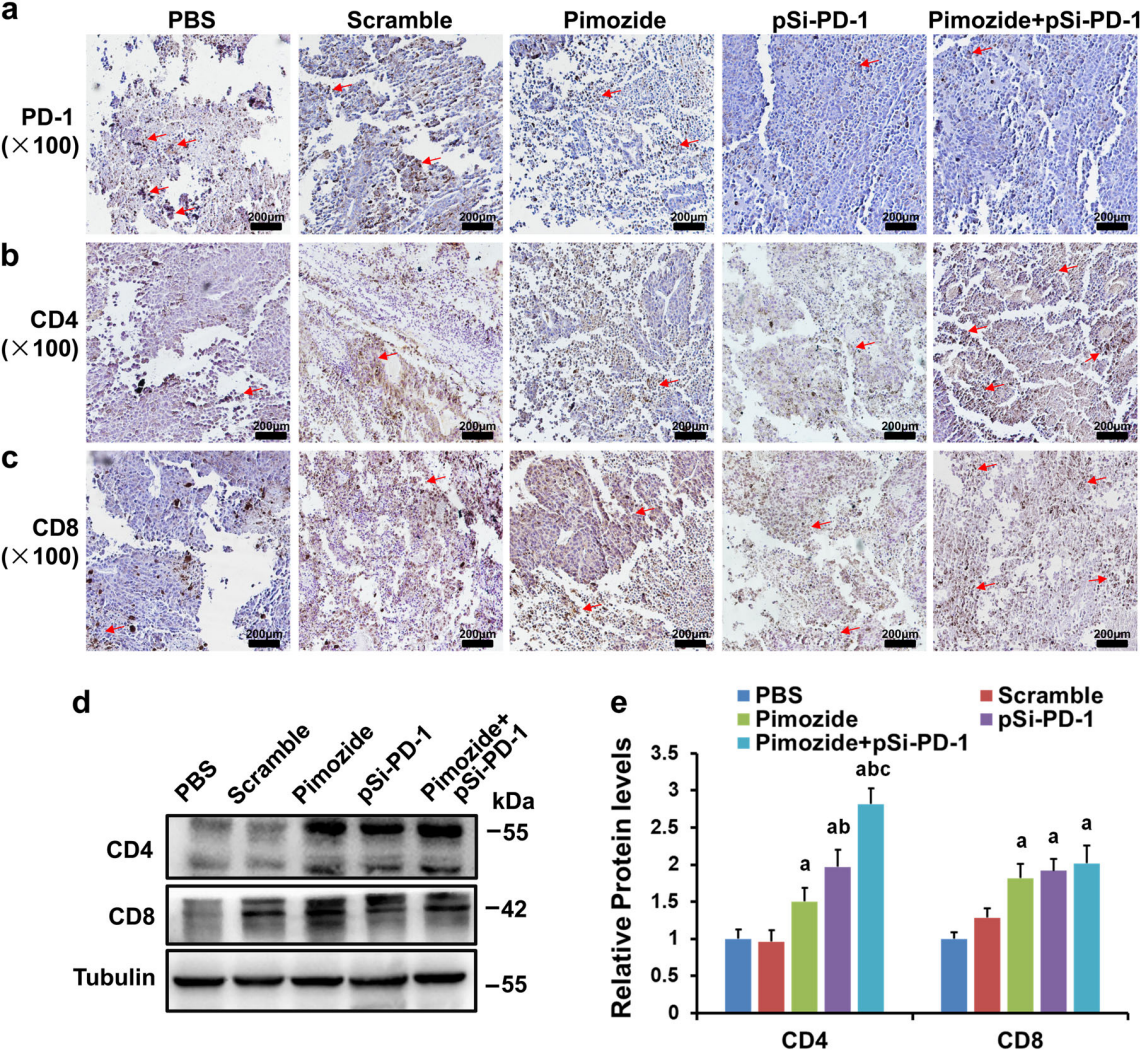
Tumor-infiltrating T lymphocyte analyses. Programmed death 1 (PD-1) expression (**a**) and the recruitment of CD4^+^ (**b**) and CD8^+^ (**c**) T lymphocytes in tumor tissues analyzed by immunohistochemistry (IHC). **d** CD4 and CD8 protein expression levels in tumor tissues detected using western blotting (WB). **e** Semiquantitative analysis of CD4 and CD8 levels in each group. ^a^*P* < 0.05 vs the Mock or pSi-Scramble group. ^b^*P* *<* 0.05 vs the pimozide group. ^c^*P* < 0.05 vs the pSi-PD-1 group. The arrows point to IHC-positive cells

To determine the potential mechanism of cell immunity in xenograft tumors, we analyzed the effects of the combination therapy on intratumor lymphocyte infiltration. We first examined PD-1, CD4, and CD8 protein levels using immunohistochemistry (IHC) assays. Figure 5a shows that PD-1 levels were considerably lower in the pSi-PD-1 and combination groups due to the presence of PD-1 siRNA. Figure 5b, c shows that both CD4^+^ and CD8^+^ T lymphocytes were increased in tumor tissues. In addition, WB confirmed that CD4 expression was markedly elevated in the three treatment groups compared to the PBS and Scramble groups (Fig. 5d, e). Meanwhile, compared to the PBS group, the other four groups, including the pSi-Scramble group, showed a strong increase in CD8 expression (Fig. 5d, e).

### The combination therapy regulated the response of various immune cells in the spleen

**Fig. 6:**
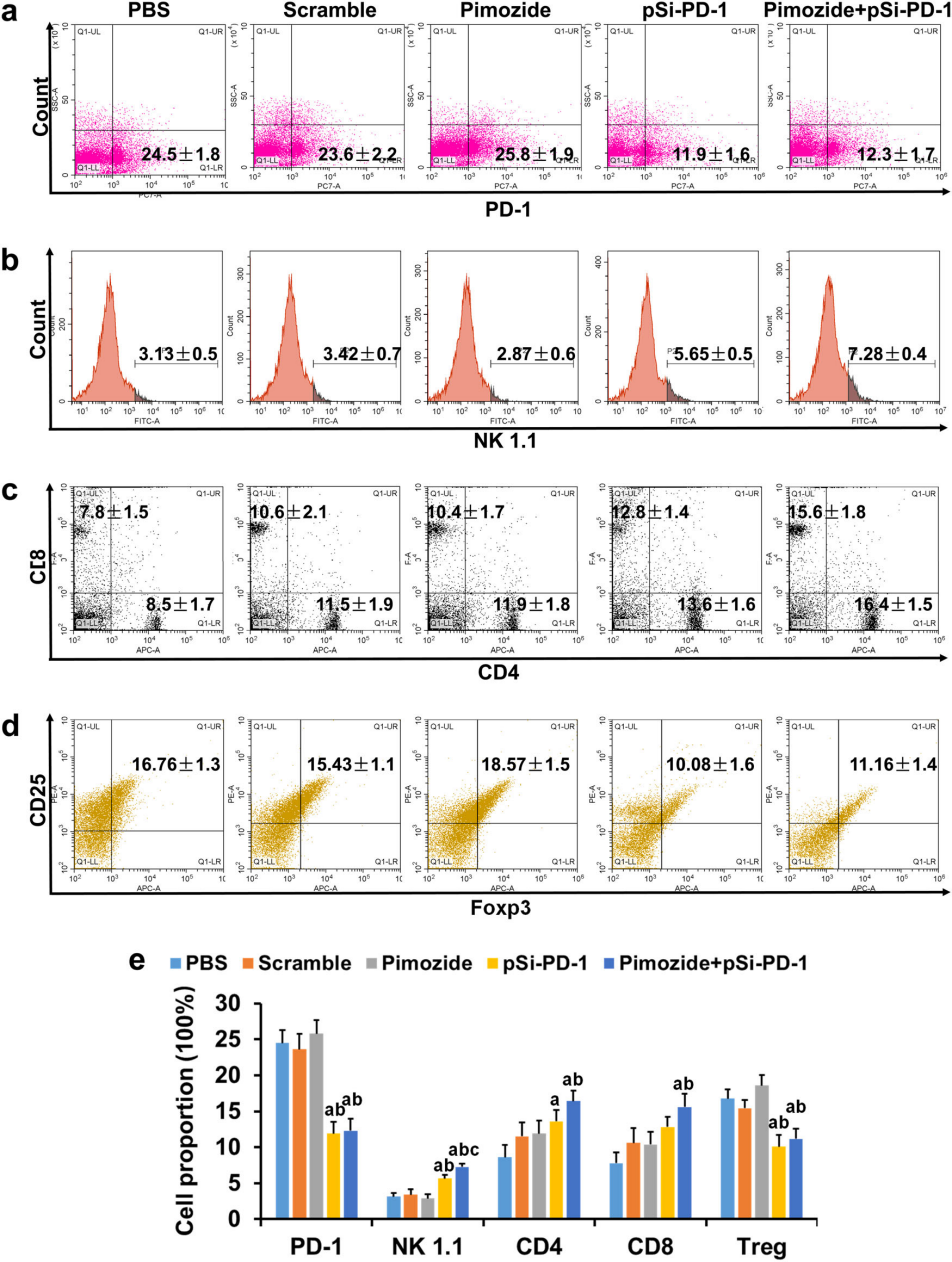
The proportion of immune cells in the spleen after various treatments. At 2 weeks after treatment, the spleen was excised, and the immune cells were evaluated using flow cytometry. Proportions of programmed death 1 (PD-1)-positive cells (**a**), NK1.1 cells (**b**), CD4^+^ and CD8^+^ T lymphocytes (**c**) and CD25^+^Foxp3^+^Treg cells (**d**). **e** The average percentages of CD4^+^ and CD8^+^ T lymphocytes, NK cells, and regulatory T cells (Tregs) were determined using statistical analysis. ^a^*P* < 0.05 vs the Mock or pSi-Scramble group. ^b^*P* *<* 0.05 vs the pimozide group. **c***P* < 0.05 vs the pSi-PD-1 group

Since the spleen plays an important role in antitumor immunity, we analyzed whether cell immunity was modulated by the combination treatment. First, we detected PD-1 expression in T lymphocytes and found that it was significantly decreased in the pSi-PD-1 and combination groups (Fig. 6a). Furthermore, we evaluated several important surface markers on T lymphocytes whose regulation may be involved in tumor growth inhibition. As shown in Fig. 6b, c, e, the proportions of CD4^+^ and CD8^+^ T lymphocytes and natural killer (NK) cells were remarkably increased in the combination therapy group compared with the PBS, pSi-Scramble, and two single treatment groups. Quantitative analysis of the flow cytometry results showed a pronounced decrease in the number of CD25^+^Foxp3^+^Treg cells in both the pSi-PD-1 and combination treatment groups compared to the PBS, pSi-Scramble, and pimozide groups (Fig. 6d, e). These results indicated that inhibition of PD-1 expression enhanced tumor immunity partly through regulation of the host immune response.

## Discussion

Decades of cancer research have centered on the molecular mechanisms that lead to cancer progression and have resulted in the development of numerous anticancer drugs that target specific aspects of the tumor microenvironment, for instance, manipulating local immunosuppression by tumors. Therefore, targeting immune checkpoint molecules is expected to become a very effective antitumor strategy. Currently, targeting PD-1 and its ligand programmed cell death 1-ligand 1 (PD-L1) to block their signaling has achieved great success in the treatment of melanoma^12,30–34^. The PD-1/PD-L1 axis plays a key role in host immune monitoring and tumor microenvironment regulation, and hence inhibiting this pathway may release immune-responsive molecules and generate a long-lasting antitumor response in combination with anticancer drugs^35,36^. However, tumor development and progression are complicated, and monotherapy usually fails to meet the demands of clinical treatment. Therefore, a number of clinical and experimental efforts have focused on combinations of standard chemotherapy, radiotherapy, and immunotherapy to treat cancer^37–40^. Therefore, we aimed to investigate the effect of the combined application of the small molecule pimozide and pSi-PD-1 on tumor cells in a mouse model of melanoma.

In this study, we first constructed the therapeutic vector harboring pSi-PD-1 and used attenuated *Salmonella* to deliver this vector to tumors. This study demonstrated that phoP/phoQ-deleted *S. Typhimurium Salmonella* performed the targeted delivery of therapeutic shRNA to tumor cells. We also found that monotherapy with pimozide or pSi-PD-1 suppressed tumor growth in B16 tumor-bearing mice. However, the combined application of pimozide and pSi-PD-1 significantly inhibited tumor growth in B16 tumor-bearing mice and prolonged survival, indicating that inhibiting PD-1 expression enhances the antimelanoma effect of pimozide. Additionally, tumor suppressive activity was observed in the pSi-Scramble group due to the antitumor activity of attenuated *Salmonella*, which carried the pSi-Scramble plasmid; this finding was consistent with our previous finding^29^.

Furthermore, we explored the underlying mechanisms of the combination therapy to improve its therapeutic effects. Caspase 3 is the key enzyme that activates apoptosis by both the extrinsic (death ligand) and intrinsic (mitochondrial) pathways under both physiological and pathological conditions^41^. We found high cleaved caspase 3 levels in the pimozide and pSi-PD-1 combination group compared with the other four groups, indicating that the combination therapy induced greater apoptosis of B16 cells. These findings illustrated that caspase 3-mediated apoptosis was one of the important mechanisms by which pimozide combined with pSi-PD-1 suppressed tumor growth.

Immune tolerance is one of the most important factors for the long-term viability of malignant tumors in vivo. In symptomatic cancer patients, T cells in the tumor microenvironment usually express PD-1, and the interaction between PD-1 and PD-L1 evokes a network that blocks T cell-mediated cancer eradication and results in tumor immune escape. In addition to T cells, both NK cells and other immune cells have been shown to express PD-1^42^. As expected, PD-1 expression was significantly decreased in the pSi-PD-1 monotherapy and combination treatment groups in the spleen and tumor tissue. Due to the presence of siRNA targeting PD-1 expression, the number of CD4^+^ and CD8^+^ T lymphocytes and NK cells was significantly increased in the spleen. In addition, the number of CD4^+^ and CD8^+^ T lymphocytes in tumor tissues from mice in the combination treatment group was also significantly increased, as shown by IHC and WB. Immune stimulation was observed in the pimozide monotherapy group, which was consistent with the results of our previous work^6^. The number of regulatory T cells (Tregs), which are key components that induce the immune tolerance of cancer cells, also increased in the spleen of mice on combination therapy. Although the mechanism is not entirely clear, the stimulation of immune cells, namely, T lymphocytes and NK cells, may be responsible for killing tumor cells in the tumor microenvironment. These analyses, though only a snapshot on day 7 after the last treatment, highlight the immune stimulation by the combination treatment, with evident regulation of immune cell populations in the spleen and within the tumor.

**Fig. 7:**
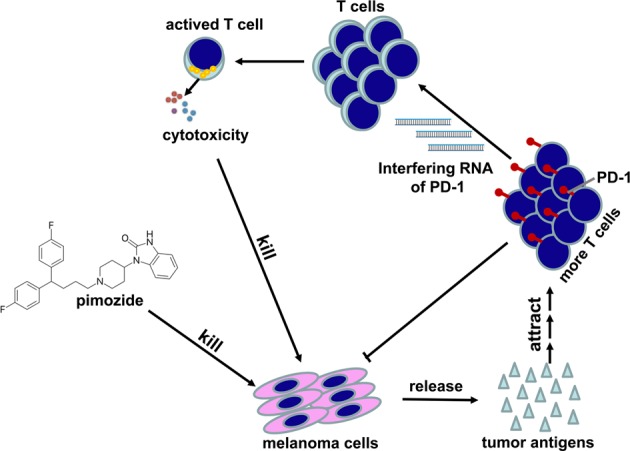
Overview of the synergistic antimelanoma effect of pimozide and pSi-PD-1 in combination. Pimozide kills tumor cells, thereby releasing more antigens and attracting more T cells to the tumor, and the addition of programmed death 1 (PD-1)-small interfering RNA (siRNA) interferes with PD-1 expression on the surface of T cells, thereby restoring the killing function of T cells and playing a synergistic antitumor role

This study demonstrated that pimozide showed synergistic effects with pSi-PD-1 delivered by attenuated *Salmonella*. The mechanism is based partly attributed to tumor cell killing by pimozide, which releases more antigens and attracts more T cells to the tumor. The inclusion of PD-1-siRNA, which interferes with PD-1 expression on the surface of T cells, restores the killing function of T cells and thus plays a synergistic antitumor role (Fig. 7). The study results suggested that immunotherapy based on targeting PD-1 may be an appropriate and clinically applicable anticancer strategy when combined with chemotherapy.

## Materials and methods

### Plasmid construction and bacteria

According to the design principles, three suitable sites target against PD-1 were selected and the specificities were determined by BLAST searches. The three sequences of PD-1-specific hairpin RNA are given as follows: 1. GATCCGGGTTTGAGCCAACCCGTCCAG*TTCAAGAGA*CTGGACGGGTTGGCTCAAACCTTTTTTGGAAA, 2. GATCCGGCCGGTTTCAAGGCATGGTCA*TTCAAGAGA*TGACCATGCCTTGAAACCGGCTTTTTTGGAAA, 3. GATCCGGACATGAGGATGGACATTGTT*TTCAAGAGA*AACAATGTCCATCCTCATGTCTTTTTTGGAAA. A Scrambled siRNA was used as a negative control. The complementary oligonucleotides were annealed and ligated into the linearized vector of pGCsilencerU6/Neo, as described previously^43^. The constructed recombinant plasmids were designated pSi-PD-1-1, pSi-PD-1-2, pSi-PD-1-3. The attenuated *S. typhimurium phoP/phoQ-*null strain LH430 was created from *S. typhimurium* strain SL1344 by deletion of the *attenuated S. typhimurium phoP/phoQ null strain LH430* locus^44^. The recombinant plasmids were electroporated into the Salmonella before use.

### Cell culture and transfection

The B16 murine melanoma cell line was obtained from Professor Liying Wang (Department of Molecular Biology, Jilin University, Changchun, China). The EL4 cell line was purchased from American Type Culture Collection (ATCC, Rockville, MD, USA). The cells were cultured in RPMI-1640 (HyClone; GE Healthcare Life Sciences, Logan, UT, USA) with 10% fetal bovine serum (MP Biomedicals, LLC, Santa Ana, CA, USA) under the following conditions: 5% CO_2_, 37 ℃, and the presence of a supersaturated solution of copper sulfate in the incubator. Cell transfection was performed using Lipofectamine 2000 (Invitrogen, Carlsbad, CA, USA) according to the manufacturer’s instructions.

### Mice and reagents

The 6-week-old male C57BL/6 mice (specific-pathogen-free grade) were obtained from Beijing Vital River Laboratory Animal Technology Co., Ltd (Beijing, China) and maintained at 25 ± 2 °C with a 12 h light/dark cycle under pathogen-free conditions. The mice had free access to food and water, and the animal studies were approved by the Ethics Committee of Xinxiang Medical University (Xinxiang, China). Pimozide was purchased from Shanghai ZZBIO Co., Ltd (Shanghai, China).

### Western blot

Western blot was performed as described before^6^. The whole cell extracts were prepared with lysis buffer. The protein concentrations were determined using a bicinchoninic acid protein assay (Beyotime Institute of Biotechnology). Protein samples (50 μg/lane) were then separated by sodium dodecyl sulfate-polyacrylamide gel electrophoresis on 12% resolving gels and transferred to polyvinylidene fluoride membranes (EMD Millipore, Billerica, MA, USA). The membranes were blocked with 5% non-fat milk for 1 h at room temperature and then incubated with the following primary antibodies overnight at 4 ℃: PD-1 (1:1000), p-Stat5 (1:1000), Stat5 (1:1000), cleaved Caspase 3 (1:1000), CD4 (1:1000), CD8 (1:1000), and Tubulin (1:1000). All antibodies were purchased from Cell Signaling Technology, Inc. Horseradish peroxidase-conjugated anti-rabbit or anti-mouse immunoglobulin G secondary antibodies (1:2000; Cell Signaling Technology, Inc.) were used for 1 h at room temperature. Specific immune complexes were visualized using enhanced chemiluminescence (Beyotime Institute of Biotechnology). The results of western blot were semi-quantified with Quantity One software (Version 4.62; Bio-Rad Laboratories, Inc., Hercules, CA, USA).

### Establishment of tumor model and treatment

Melanoma-bearing mice were established via the subcutaneous (s.c.) inoculation of B16 cells. Briefly, 1 × 10^6^ B16 cells were injected (s.c.) into the right flank of C57BL/6 mice. At 7 days after tumor inoculation, the mice were randomly divided into five groups. Mice in the PBS group received daily i.p. injections of 100 μl PBS for 1 week. Mice in the pimozide group received daily i.p. injections of 200 μg pimozide for 1 week. Mice in the pSi-Scramble group were injected intratumorally with recombinant attenuated *Salmonella* harboring pSi-Scramble (2 × 10^6^ colony-forming units (CFU) in 100 μl PBS/mouse) twice on days 7 and 14. Mice in the pSi-PD-1 group were injected intratumorally with recombinant attenuated *Salmonella* harboring pSi-PD-1 (2 × 10^6^ CFUs in 100 μl PBS/mouse) twice on days 7 and 14. Mice in the combination treatment group received daily i.p. injections of 200 μg pimozide for 1 week and intratumoral injections of recombinant attenuated *Salmonella* harboring pSi-PD-1 (2 × 10^6^ CFUs in 100 μl PBS/mouse) twice on days 7 and 14.

### Colony formation assays

Tissue samples (100 mg) were taken from the heart, liver, spleen, lungs, kidneys, and tumors of experimental mice under aseptic conditions. The tissue was ground and added to 3 ml cold PBS. Then, 300 μl aliquots of resuspended tissue were mixed with 700 μl PBS. The mixture was inoculated onto solid LB plates containing ampicillin (final concentration, 100 μg/ml) and cultured overnight at 37 ℃. The number of bacterial colonies formed on the next day was counted and analyzed quantitatively.

### Immunohistochemical staining

The immunostaining of CD4, CD8, and PD-1 proteins in murine melanoma tissues was performed according to our previously published protocol^6^. Sections were deparaffinized and dehydrated in a series of xylene and alcohol washes. Then, antigen retrieval was performed by heating the tissue sections in a microwave for 10 min in a citrate solution (10 mmol/l, pH 6.0).The tissues were then blocked with 1% (wt/vol) bovine serum albumin (Gibco; Thermo Fisher Scientific, Inc., Waltham, MA, USA) at room temperature for 15 min and incubated with monoclonal antibodies directed against CD4 (1:100), CD8 (1:100), and PD-1 (1:100) (Cell Signaling Technology, Inc., Danvers, MA, USA) overnight at 4 ℃. The sections were rinsed by PBS for 5 min and blocked with a horseradish peroxidase-conjugated immunoglobulin G secondary antibody (1:1000; Bioworld Technology, Inc., St. Louis Park, MN, USA) for 30 min at room temperature. The immunostaining intensities of CD4, CD8, and PD-1 were assessed based on the 0 to 3+ scale as depicted before: 0, no staining identified; 1+, <25% of positive cells; 2+, 25–75% positive cells; and 3+, >75% positive cells.

### Terminal deoxynucleotidyl transferase dUTP nick end-labeling

TUNEL was performed according to the instruction (Beyotime Institute of Biotechnology). The paraffin sections were dewaxed in xylene for 10 min, replaced with fresh xylene dewaxed for 10 min, followed by anhydrous ethanol for 5 min, 95% ethanol for 5 min, 90% ethanol for 5 min, 80% ethanol for 5 min, 70% ethanol for 5 min, and distilled water for 2 min. Then tissues were covered with 20 μg/ml protease K (DNase free), incubated at 37 °C for about 20 min, and washed with PBS three times for 10 min. Each tissue sample was added with TUNEL detection solution and incubated without light at 37 °C for 60 min and washed with PBS three times for 10 min. TUNEL-positive cells were detected under fluorescence microscope.

### Flow cytometry

After treatment for 7 days, spleens were removed in each group. Spleens were grinded and lysed using Red Blood Cell Lysis Buffer (Beyotime Institute of Biotechnology), centrifuged for 5 min at 4 °C, and washed with PBS. Cell suspension was then prepared at the concentration of 1 × 10^7^/ml. Subsequently, the cell suspension of 100 µl was incubated with the appropriate fluorochrome-labeled CD3, CD4, CD8, PD-1, and NK 1.1 antibodies (all from BioLegend, Inc., Santiago, USA) in the dark at 4 °C for 30 min. The mouse regulatory T cell staining kit (Affymetrix eBioscience; Thermo Fisher Scientific, Inc.) was used to detect Tregs according to the instruction. Flow cytometry (Guava easyCyte; EMD Millipore) was used to measure the fluorescence intensity of cells with a minimum of 10,000 cells.

### Statistical analysis

All values are expressed as the means ± SD of three independent experiments. Two-tailed unpaired Student’s *t*-test, one-way analysis of variance, Mann–Whitney *U*-test, and log-rank test were performed as appropriate. A value of *P* < 0.05 was considered significant.
